# Supplementary material for: Development of a multiplexed targeted mass spectrometry assay for LRRK2-phosphorylated Rabs and Ser910/Ser935 biomarker sites
Source: Biochem J. 2021 Jan 22;478(2):299–326. doi: 10.1042/BCJ20200930 (PMC7833208; doi:10.1042/BCJ20200930)

Supplementary figure Legends

Supplementary Figure 1: **S-1A)** Equimolar ratio of the indicated pRab peptides are spiked into 50 ng of HeLa cell tryptic digest and analysed using EvoSep 21 min run gradient on QE HF-X MS. Extracted ion chromatograms depicting the separation of pRab peptides on a 21 min run gradient. pRabs are well separated between 7 to 8.5 retention time including the isobaric pRab3 and pRab35 that are well separated by 1 min apart indicated with blue coloured asterisk (*).**S1B-C)**) The retention times reproducibility was superior on 21 min run EvoSep LC system. 10fmol (S1-B) or 50fmol (1S-1C) pRabs measured retention times depicted as a linear regression curve showing an r value closer to 1. **S1-D**) 50 fmol of pRab10 synthetic peptide was analysed using 21 min on EvoSep LC system and 45 min on Dionex RSLC nano LC system. Data was acquired in a PRM mode on QE HF-X instrument. The data was analysed using Skyline software and the relative pRab10 intensity was depicted as a bar graph. Y-axis indicating the pRab10 intensity and x-axis showing the data acquired on EvoSep and Dionex LC systems. **S1-E**) the data acquired in S1-D was assessed for the carryover between the two LC-systems by measuring the pRab10 intensities in blank runs. Carryover appeared to below 0.01% on EvoSep LC system. **S1-F)** Sensitivity of the Pan-pRab antibody was assessed at a varied amounts ranging between 0.001 µg to 1 µg. 100 fmol of pRabs heavy labelled peptide mixture was spiked in to 200 µg of BSA digest and Immunoprecipitation was undertaken with an indicated antibody amounts. Bar graph depicting the relative pRabs intensities on y-axis for each of the pRab peptide on x-axis. The varied amount of antibody was highlighted in a light to dark red coloured gradient. n=3, Error bars representing the mean ±SEM.

Supplementary Figure 2: Limit of detection and quantification of targeted pRab8 and pRab10 peptides: S2-A) Immunoblotting Validation of Rab8-KO and Rab10-KO A549 cells used in Figure 2A-2B to assess the limit of detection. **S2B-S2C)** Limit of detection experiments were carried out by spiking synthetic light phosphorylated Rab8A (Thr72) peptide at (0.01, 0.1 1, 10, 50, 100, 500, 1000 fmol), while keeping the constant 50 fmol of heavy phosphorylated Rab8A (Thr72) peptide in 50ng of HeLa peptide digest. Immunoprecipitations were undertaken with Pan-pRab antibody and analysed on Orbitrap HF-X MS in a targeted PRM mode. XIC were generated using Skyline software. X-axis depicting the expected (L/H) ratio and Y-axis depicting measured (L/H) ratio. Rectangular box indicates the zoom in of 0.01, 0.1 and 1 fmol data points. n=3. **S2-B)** similar experiments were carried out for Human specific LRRK2 peptides. Error bars represent the mean ±SEM. **S2: D-F)** as described in Figure 2C-2E, similar experiments were carried out for Human specific LRRK2 peptides. The relative pRabs and pLRRK2 and total LRRK2 peptides in ach round of IP was plotted as bar graphs. X-axis representing the pRabs or LRRK2 and Y-axis representing the peptide recovery percentage. Similar experiments were undertaken by spiking 1 fmol (S2-D), 10 fmol (S2-E) and 100 fmol (S2-F) amounts. pRab43 recovery was found to be ~70% and ~30% in IP1 and IP2. n=3, error bars representing mean ±SEM

Supplementary Figure 3: **Epitope mapping of LRRK2 UDD1, UDD2, and LRRK2 C-terminal N241a/34 antibodies**. **S3-A)** Wild type A549 cells were lysed, digested with trypsin and subjected to with the indicated antibodies. Eluted peptides were analysed by MS in data

dependent mode and intensities of detected peptides represented on a heat map blue (lowest) to red (highest). **S3-B)** an orthogonal validation of 8G10 antigen sequence by PEP preprint microarray-based technology. Custom prepared LRRK2 sequence peptide microarray was incubated with 1:1000 dilution of 8G10 antibody. Spot identification and quantification was carried out using LI-COR Odyssey imaging system. Highest intensity was observed for the LRRK2 sequence (DEDGHFP LRRK2 391-397). **S3-C)** Sequence alignment of LRRK2 pS910, LRRK2pS935 and total LRRK2 peptide sequences depicting the changes between mouse (highlighted in red coloured text) and Human (highlighted in blue coloured text). **S3-D)** MS/MS spectrum of an identified Phosphopeptide HSNSLGPIFDHEDLLK (LRRK2 pS935) that is selectively enriched by UDD2 antibody. **S3-E)** MS/MS spectrum of a Phosphopeptide SNSISVGEFYR (LRRK2 pS910) that is selectively enriched by UDD1 antibody. **S3-F)** MEFs, MS/MS spectrum of a mouse specific LRRK2 peptide IGDEDGQFPAHR (LRRK2 388-399) that was selectively enriched by 8G10 antibody in VPS35 [D620N] MEFs.

Supplementary Figure 4: **Determining the optimal collision energies for pRabs and pLRRK2 and total LRRK2 peptides: S4: A-B)** Equimolar mixture of 10 fmol (S4-A) or 50 fmol (S4-B) containing six pRabs and LRRK2-pS910/pS935 and total LRRK2 were spiked into 50ng of HeLa peptide digest. HCD collision energies (CE) 25, 27 and 30 were tested to determine the best CE fragmentation for indicated peptides. Box plots depicting (S4: A-B) log10 intensities on y-axis and tested HCD CE value of X axis. **S4-C)** Bar graphs depicting the distribution of coefficient variation (CV) shown in percentage for 10 fmol amount experiments of all tested pRabs and pLRRK2 peptides on y-axis. X-axis indicating the median CV% value for HCD25, 27 and 30 respectively. **S4-D)** Bar graphs depicting the distribution of coefficient variation (CV) shown in percentage for 50 fmol amount experiments of all tested pRabs and pLRRK2 peptides on y-axis. X-axis indicating the median CV% value for HCD25, 27 and 30 respectively. n=3, error bars representing mean ±SEM

Supplementary Figure 5: Development of total Rab proteins targeted PRM assay. S5-A) XIC depicting the 50 Fmol of an equimolar mixture of total Rab peptides (Rav1A, Rab1B, Rab3D, Rab8A, Rab8B, Rab10, Rab12, Rab35 and Rab43) was spiked into 50ng of HeLa and separated on EvoSep LC using 44 min. S5-B) XIC depicting 10 fmol of equimolar mixture of total Rab peptides separated on EvoSep LC using 21 min run. Retention times corresponding to each Rab was indicated in red coloured text (S5: A-B) and poorly separated Rab peptides in a 21 min run are indicated with orange coloured text. S5-C) Lysates of HEK293 cells overexpressing HA-Rab1A, HA-Rab1B, HA-Rab1C or HA-empty were used for HA immunoprecipitation (300 µg per IP). Immunoprecipitates were then immunoblotted with the Rab1-total and ant-HA antibodies. Immunoblots were subjected to quantitative LI-COR immunoblot analysis with all indicated antibodies at 1 µg/mL. S5: D-E) 50 fmol of heavy pRab12 spiked in 50ng of HeLa mixture was assessed to verify the immunoprecipitation efficiency using pRab12 antibody. 100Fmol of pRab12 heavy peptide was spiked into 200 µg of BSA tryptic digest and subjected to immunoprecipitation and analysed using PRM method. IP-2 indicating the flow-through was subjected to second round of Immunoprecipitation by adding fresh 1µg of pRab12 antibody. Immunoprecipitation was also assessed for samples Immunoprecipitated at room temperature

and 50°C. S5-E) Peak areas depicting for the samples analysed in S5-C. n=3, error bars representing mean ±SEM

Supplementary Figure 6: **(A-H).** MLi-2 inhibition IC_{50} values for both wild type and VPS35[D620N] were determined from the data presented in Figure 4, by dividing L/H ratio of each indicated MLi-2 concentration over the average value of L/H ratio of DMSO as 100%. The IC_{50} for each pRab was calculated using GraphPad prism software and indicated.

Supplementary Figure 7: **(A-F)** : Multiplexed PRM analysis of litter mate matched wild type and LRRK2 A2016T MEFs that are treated in a dose dependent manner of (0, 1, 3, 10, 30,100 and 300 nM) MLi2. No significant decrease of pRab1, pRab3 and pRab35 levels were observed. The bottom panel representing the total Rab protein levels. D-F) PRM analysis of total Rab levels for Rab1B, Rab8B and Rab12. n=3, error bars representing the mean ±SEM.

Supplementary Figure 8: **(A-H)**. Targeted MS analysis of Wild type and LRRK2 A2016T MEFs reveals the MLi2 IC_{50} values of pRabs and LRRK2-pS910/pS935: MLi-2 inhibition IC_{50} values for both wild type and LRRK2 (A2016T) MEFs were determined from the data presented in Figure 6, by dividing L/H ratio of each indicated MLi-2 concentration over the average value of L/H ratio of DMSO as 100%. The IC_{50} for each pRab was calculated using GraphPad prism software and indicated.

Supplementary Figure 9: **(A-B).** A) The indicated WT and LRRK2[A2016T] MEFs generated in Figure 6, were treated with or without the indicated concentrations of MLi-2 for 90 min. Cells were lysed, and 10 µg of extract was subjected to quantitative immunoblot analysis with the indicated antibodies (all at 1 µg/ml). Each lane represents cell extract obtained from a different dish of cells (3-4 replicates per condition). The membranes were developed using the Odyssey CLx Western Blot imaging system. B) Immunoblots were quantified using the Image Studio software and depicting the relative abundance of pRab10 and LRRK2-pS935. Data are presented relative to the phosphorylation ratio observed in WT cells treated with DMSO (no inhibitor), as mean ± SEM n=3.

Supplementary Figure 10: (**A-B)** 25 µg of extracts from the indicated tissues of WT and LRRK2 [R1441C] littermate mice harvested in Figure 8, were subjected to quantitative immunoblot analysis with the indicated antibodies (all at 1 µg/ml). Each lane represents cell extract obtained from a different animals (2-4 replicates per condition). The membranes were developed using the Odyssey CLx Western Blot imaging system. Immunoblots were quantified using the Image Studio software. Bottom panel of the immunoblot representing the quantification relative to the phosphorylation ratio observed in WT tissue (no ML-2). Statistical significance was determined by two tailed T-test and the significance of pRab10 (Brain: not significant P= 0.052), Kidney (ns P=0.067), Lung (P=0.0207) and Spleen (P= 0.0015). For LRRK2 pS935 (Brain, (p=<0.00001), Kidney (P=0.0004), Lungs (P=0.000104) and Spleen (p= 0.001304). n=4 for vehicle and n=2 for MLi-2 treated mice. Error bars representing the mean ±SEM.

Supplementary Figure 11: **A-B)** 25 μg of extracts from the indicated tissues of WT and VPS35[D620N] littermate mice harvested in Figure 9, were subjected to quantitative immunoblot analysis with the indicated antibodies (all at 1 μg/ml). Each lane represents cell extract obtained from a different animals (2-4 replicates per condition). The membranes were developed using the Odyssey CLx Western Blot imaging system. Immunoblots were quantified using the Image Studio software. Bottom panel of the immunoblot representing the quantification relative to the phosphorylation ratio observed in WT tissue (no ML-2). Statistical significance was determined by two tailed T-test and the significance of pRab10 (Brain (P=0.0270), Kidney (P=<0.00001), Lung (P=<0.00001) and Spleen (P=0.000271). For LRRK2 pS935 no statistical significance has been observed, (Brain, (p=0.47), Kidney (P=0.85), Lungs (P=0. 0.47) and Spleen (p= 0.93). n=4 for vehicle and n=2 for MLi-2 treated mice. Error bars representing the mean ±SEM.

Supplementary Figure 12: Targeted MS assay of LRRK2-Rab signalling pathway in human neutrophils. Primary neutrophils isolated from 3 healthy donors were treated with the indicated concentrations of MLi-2 for 60 min prior to harvest and subjected to either tryptic digestion (**A to D**) or immunoblot analysis (**E**). (**A to D**) 150 μg of digest was spiked with an equimolar ratio of 100 fmol heavy pRabs and LRRK2 pS910/pS935 and total LRRK2 peptides and subjected to sequential immunoprecipitation (IP) with a multiplexed antibody cocktail (Pan-pRab, UDD1, UDD2 and 8G10) and peptide levels quantified as fmol peptide/mg of tryptic digest (upper panel). In parallel, 0.5 μg of tryptic digest spiked with an equimolar ratio of 50 fmol of heavy total Rab peptides and data was acquired in PRM mode and peptide levels normalised to the median peptide intensity (lower panel). Dotted arrows indicated the differences between ± 300 nM MLi-2 samples and one tailed student t-Test was performed to depict the significance of change indicated with *. Three independent replicate experiments were performed for each of the indicated MLi-2 concentration and each dot indicates values for each experiment. Error bars representing mean ±SEM. (**E**) 10 μg of extract was subjected to quantitative immunoblot analysis with the indicated antibodies (all at 1 μg/ml). Each lane represents cell extract obtained from a different donors. Data are presented relative to the phosphorylation ratio observed in cells treated with DMSO (no inhibitor), as mean ± SEM. Statistical analysis was performed by one tailed student T-test indicating the significance between DMSO and 300nM MLi-2 conditions. A-D) Statistical significance represented in for PRM analysis are pRab10 (P= 0.017), pRab43 (P= 0.0024), LRRK2-pS910 (P=0.0078), LRRK2-pS935 (0.0048). G-F) Significance representing for the immunoblot analysis of pRab10 (p= 0.0034), LRRK2-pS935 (0.000556). Error bars representing the mean ±SEM.

Supplementary table legends

Supplemental table 1: Details of reagents, antibodies, cDNA clones, Knock-in mice, MEFs, Heavy synthetic peptides equipment and software used in the current study.

Supplemental table 2: Identification and quantification of Wild type MEFs proteome, Wt VPS35 and VPS35[D620N] MEFs IC-50 pRabs PRM experiments, Wt LRRK2 and LRRK2[A2016T] MEFs

IC-50 pRabs PRM experiments, Wt LRRK2 and LRRK2[R1441C] MEFs pRabs PRM experiments, Wt LRRK2 and LRRK2[R1441C] mice tissues (Whole brain, kidney, lungs and spleen) pRabs PRM experiments, Wt VPS35 and VPS35[D620N] mice tissues (Whole brain, kidney, lungs and spleen) pRabs PRM experiments, Human Neutrophils VPS35[D620N] patients and Healthy donors pRabs PRM experiments, Human Neutrophils IC-50 pRabs PRM experiments and 21 min PRM QE HF-X instrument parameters used in this study.

Supplemental table 3: Identification and quantification of Wt VPS35 and VPS35[D620N] MEFs IC-50 total Rabs PRM experiments, Wt LRRK2 and LRRK2[A2016T] MEFs IC-50 total Rabs PRM experiments, Wt LRRK2 and LRRK2[R1441C] MEFs total Rabs PRM experiments, Wt LRRK2 and LRRK2[R1441C] mice tissues (Whole brain, kidney, lungs and spleen) total Rabs PRM experiments, Wt VPS35 and VPS35[D620N] mice tissues (Whole brain, kidney, lungs and spleen) total Rabs PRM experiments, Human Neutrophils VPS35[D620N] patients and Healthy donors total Rabs PRM experiments, HCD collision energy optimization experiments, pRab8 and pRab10 limit of detection experiments and 45 min PRM QE HF-X instrument parameters used in this study.

Supplementary Figure: 1

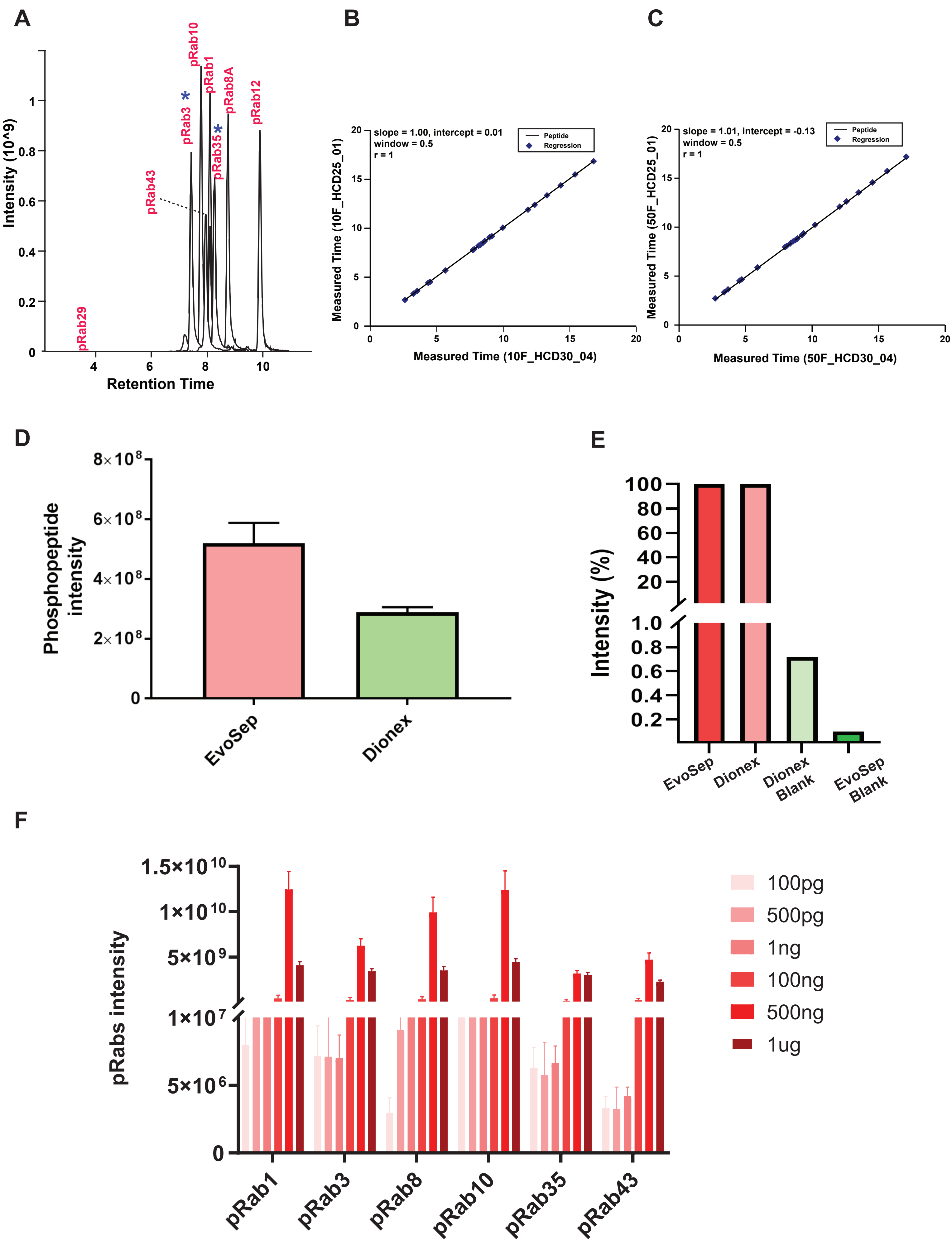

Supplementary Figure: 2

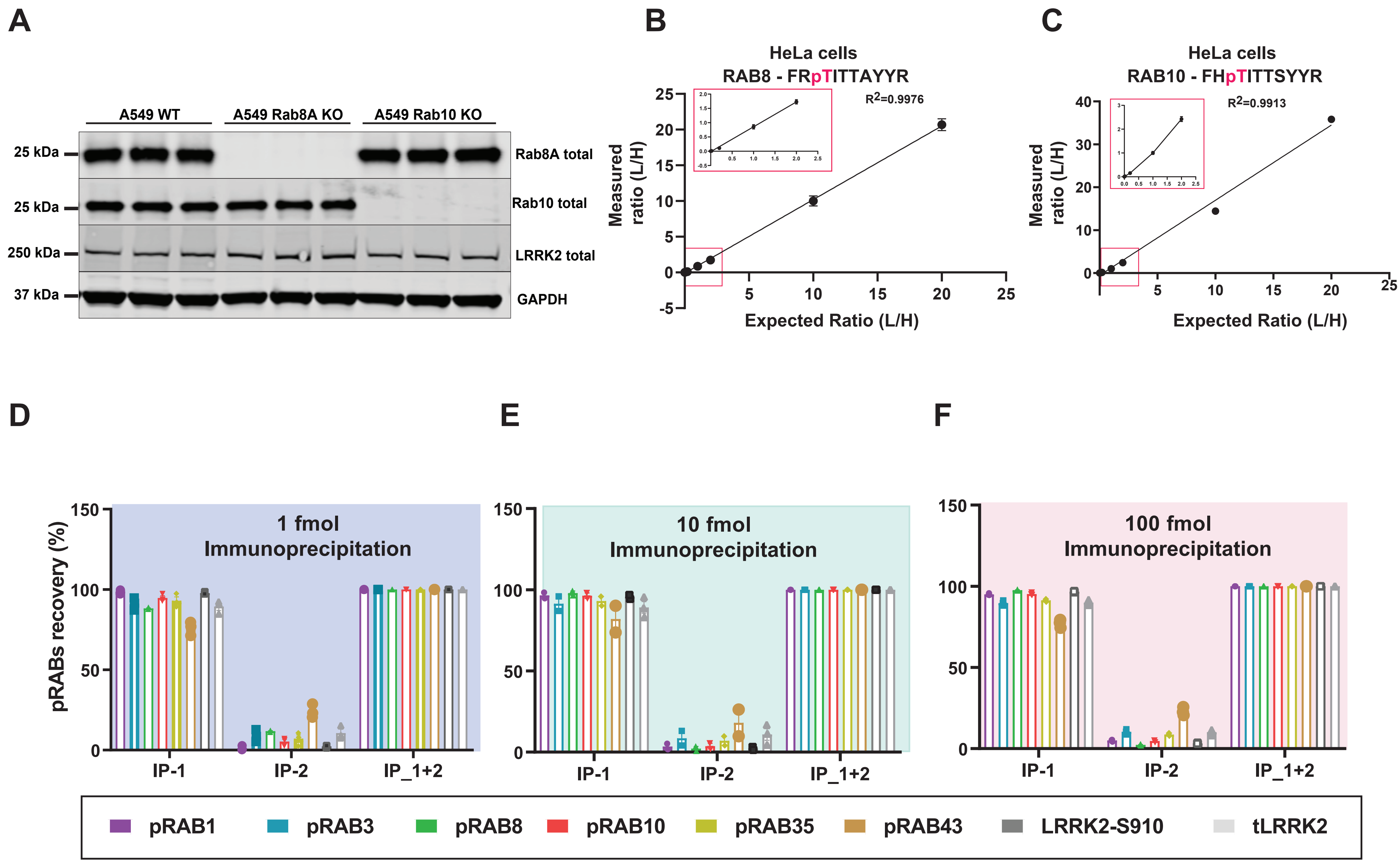

Supplementary Figure: 3

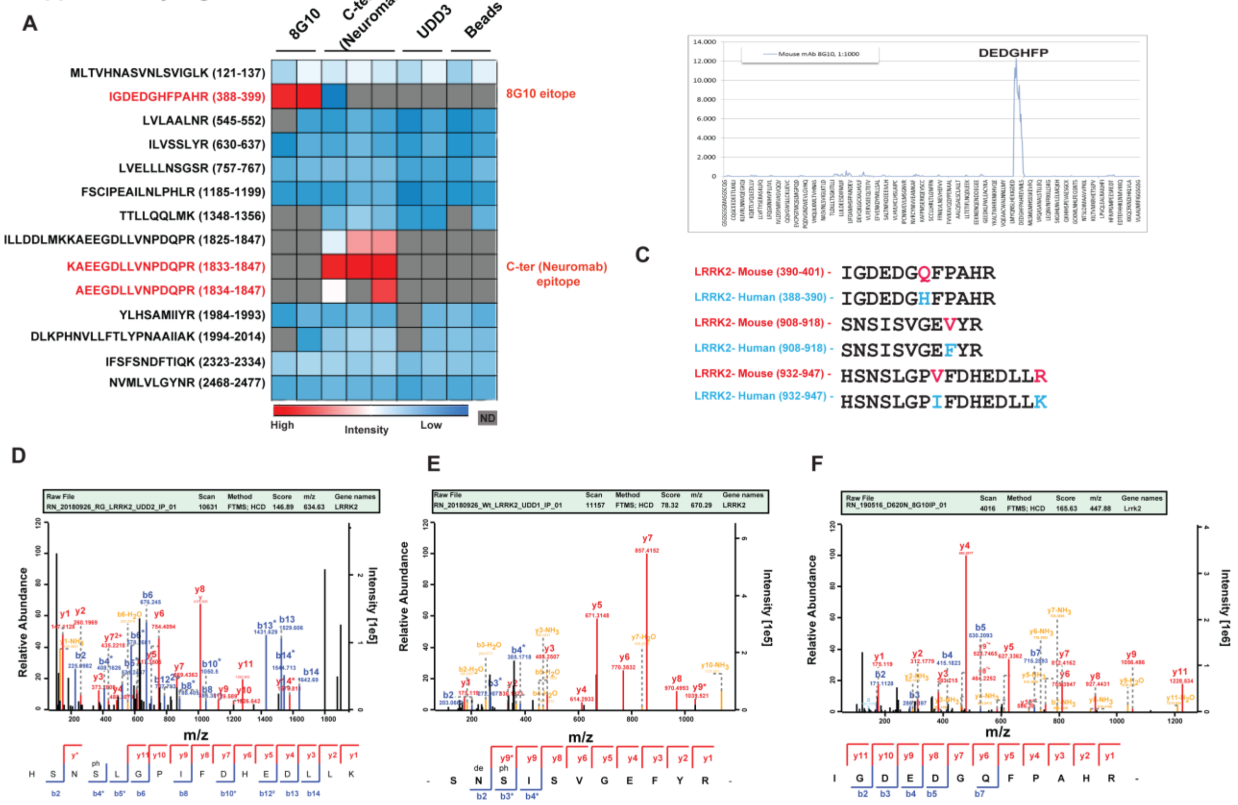

Supplementary Figure: 4

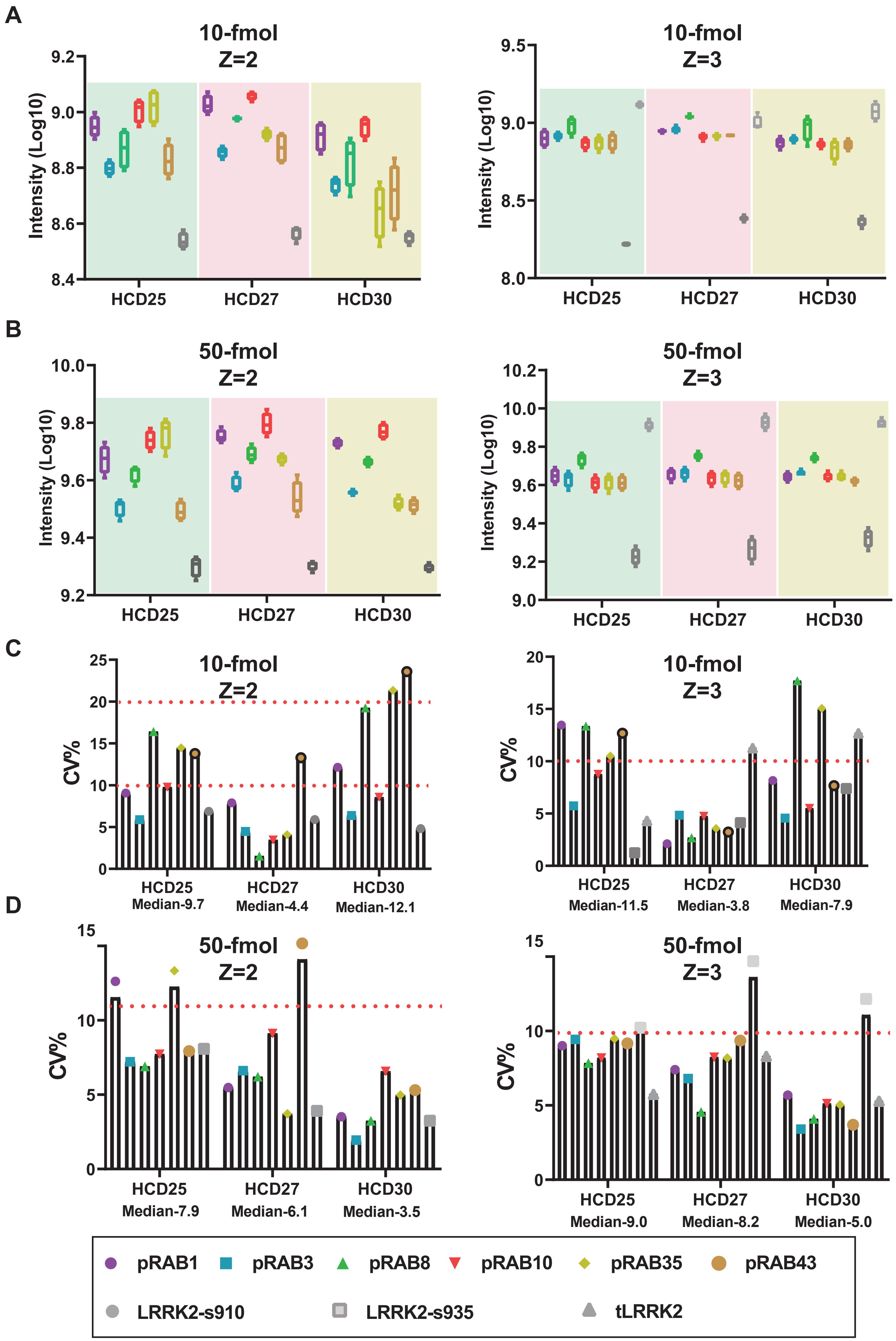

Supplementary figure:5

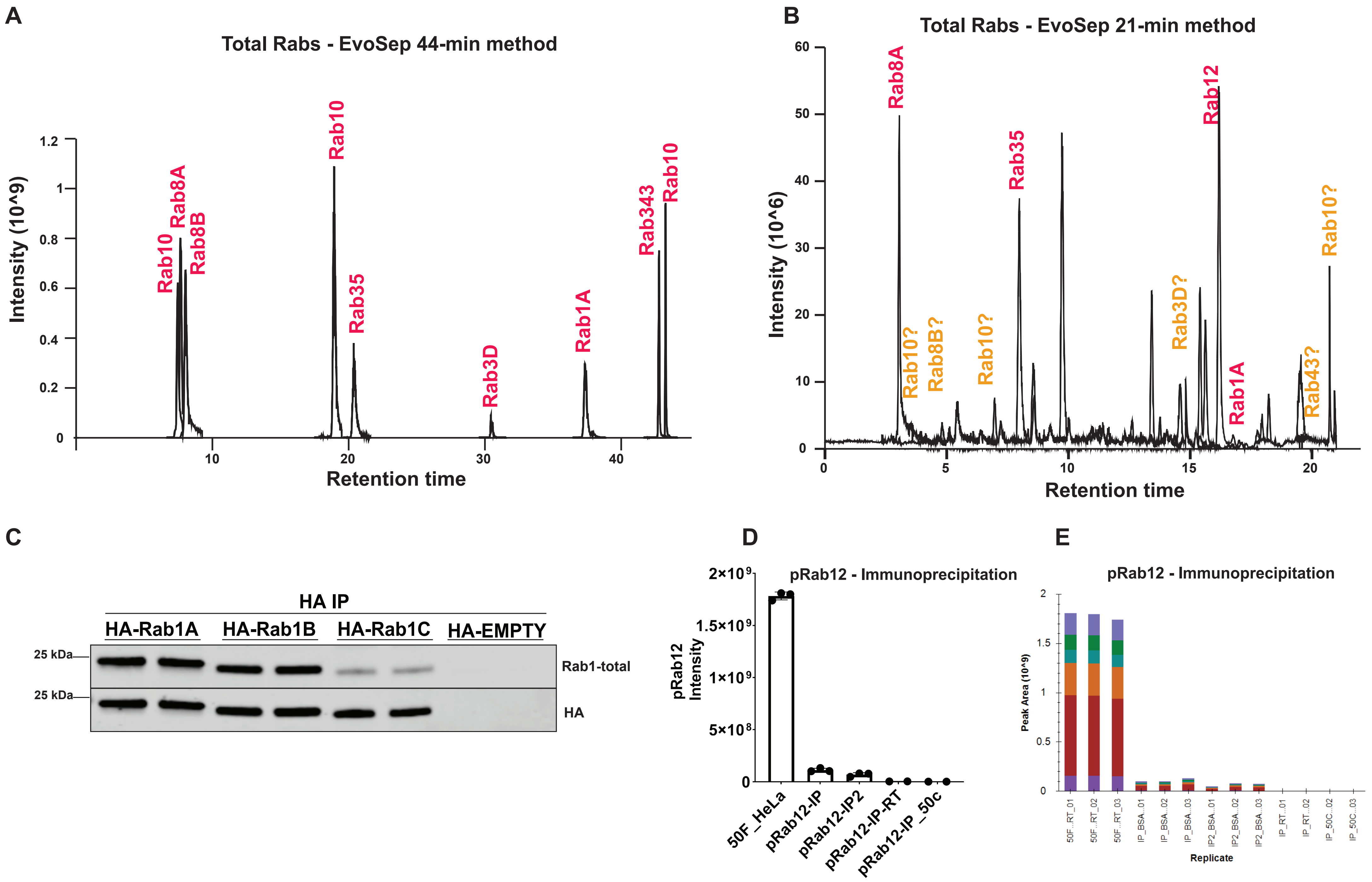

Supplementary Figure: 6

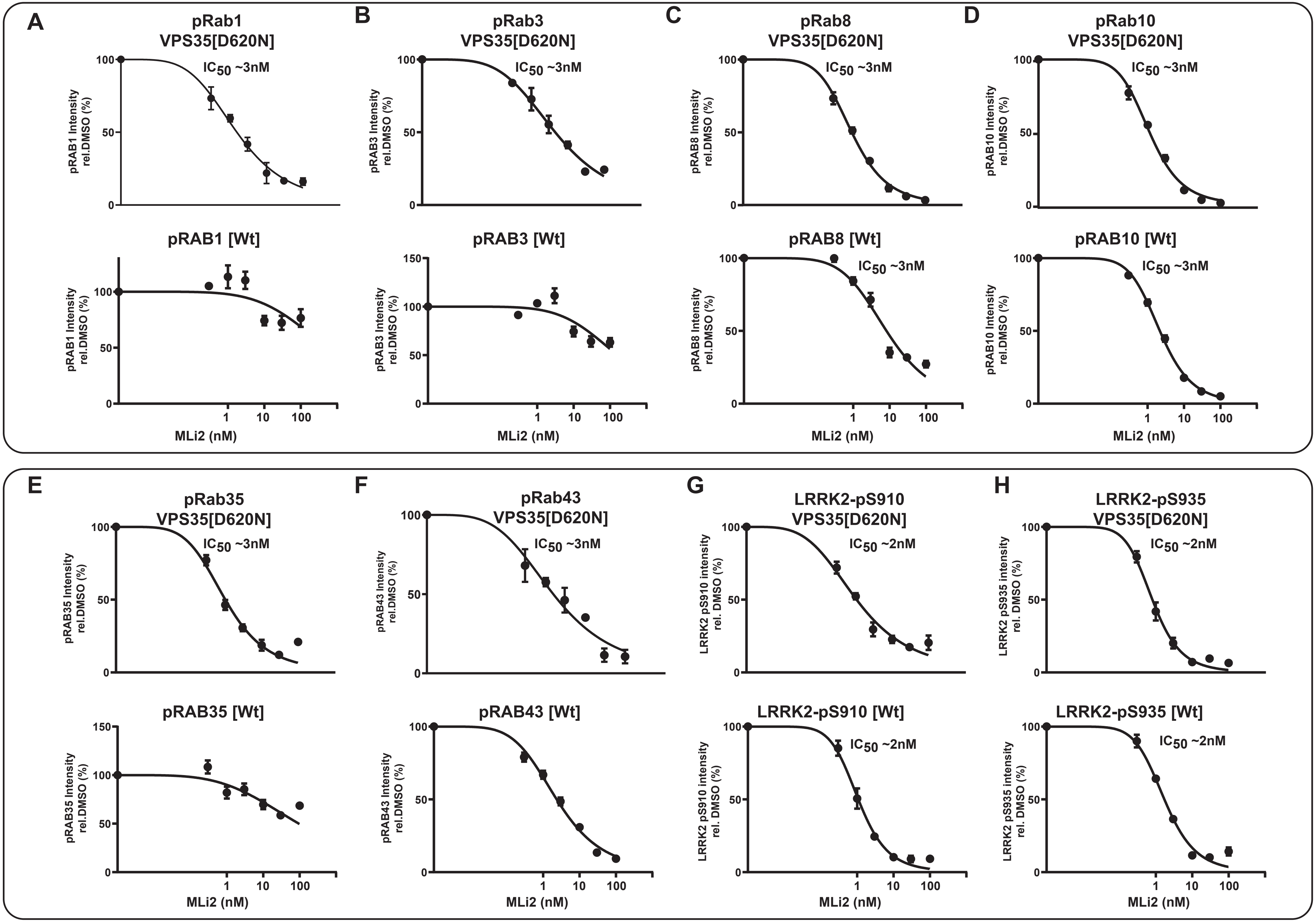

Supplementary Figure: 7

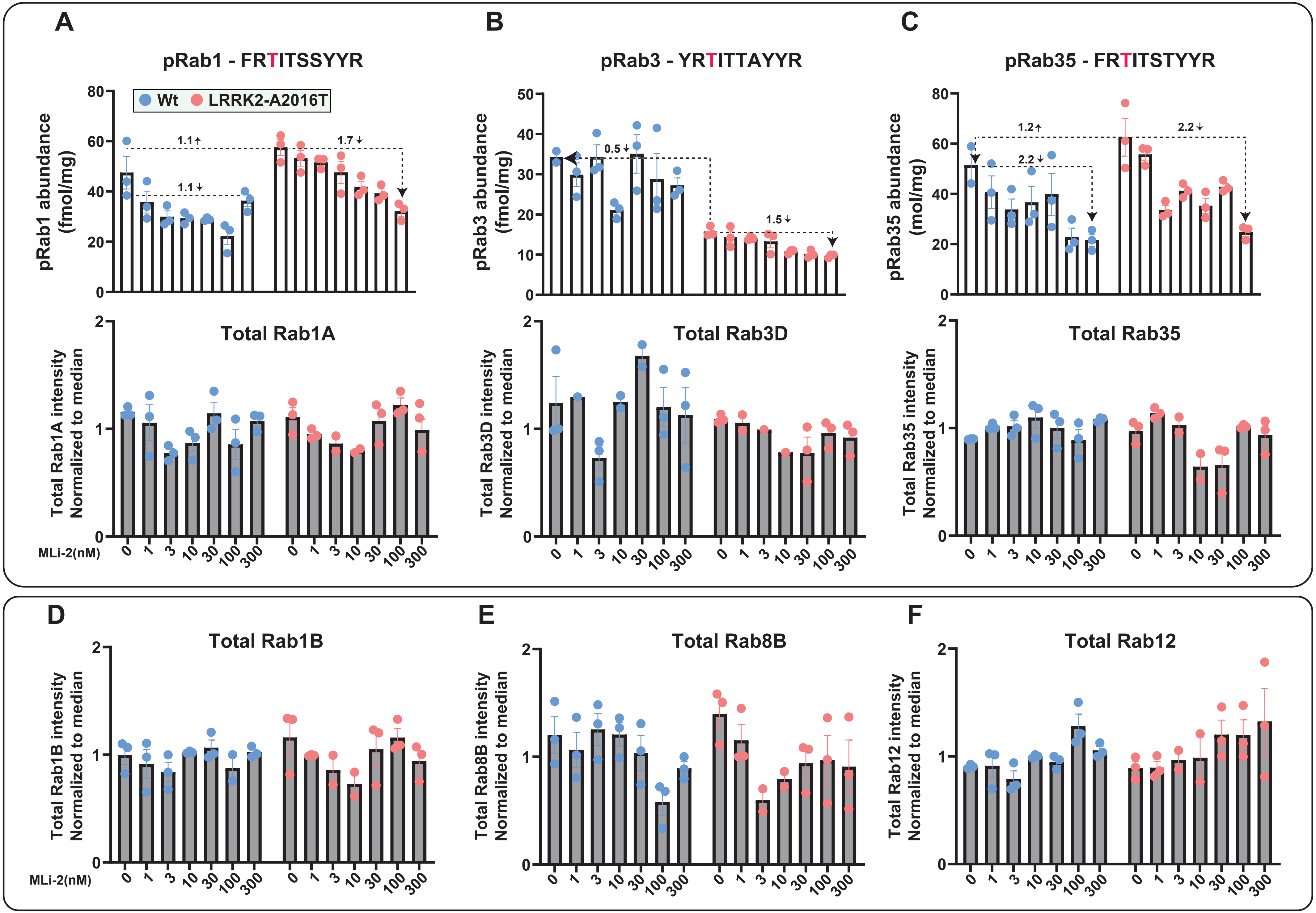

Supplementary Figure: 8

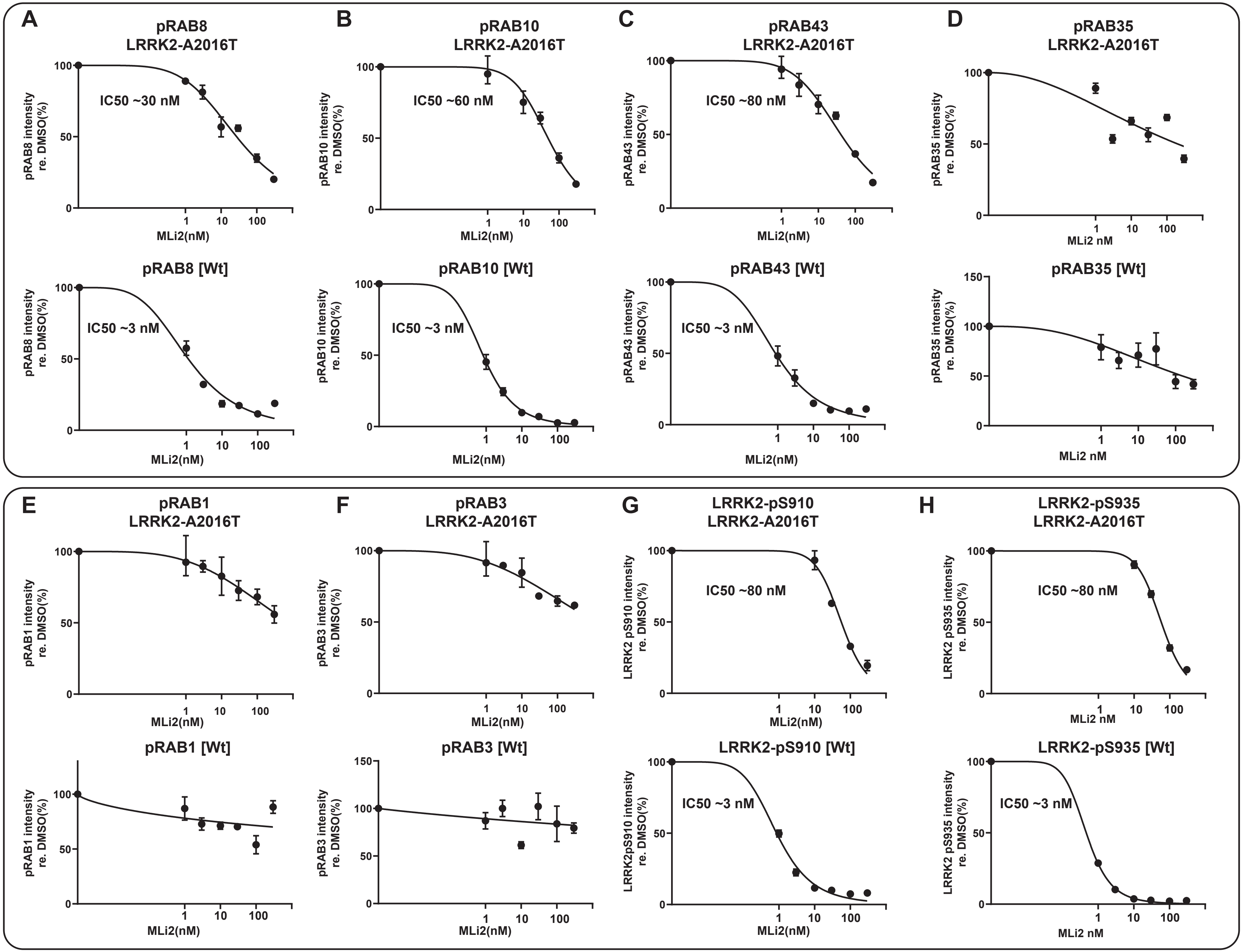

Supplementary Figure: 9

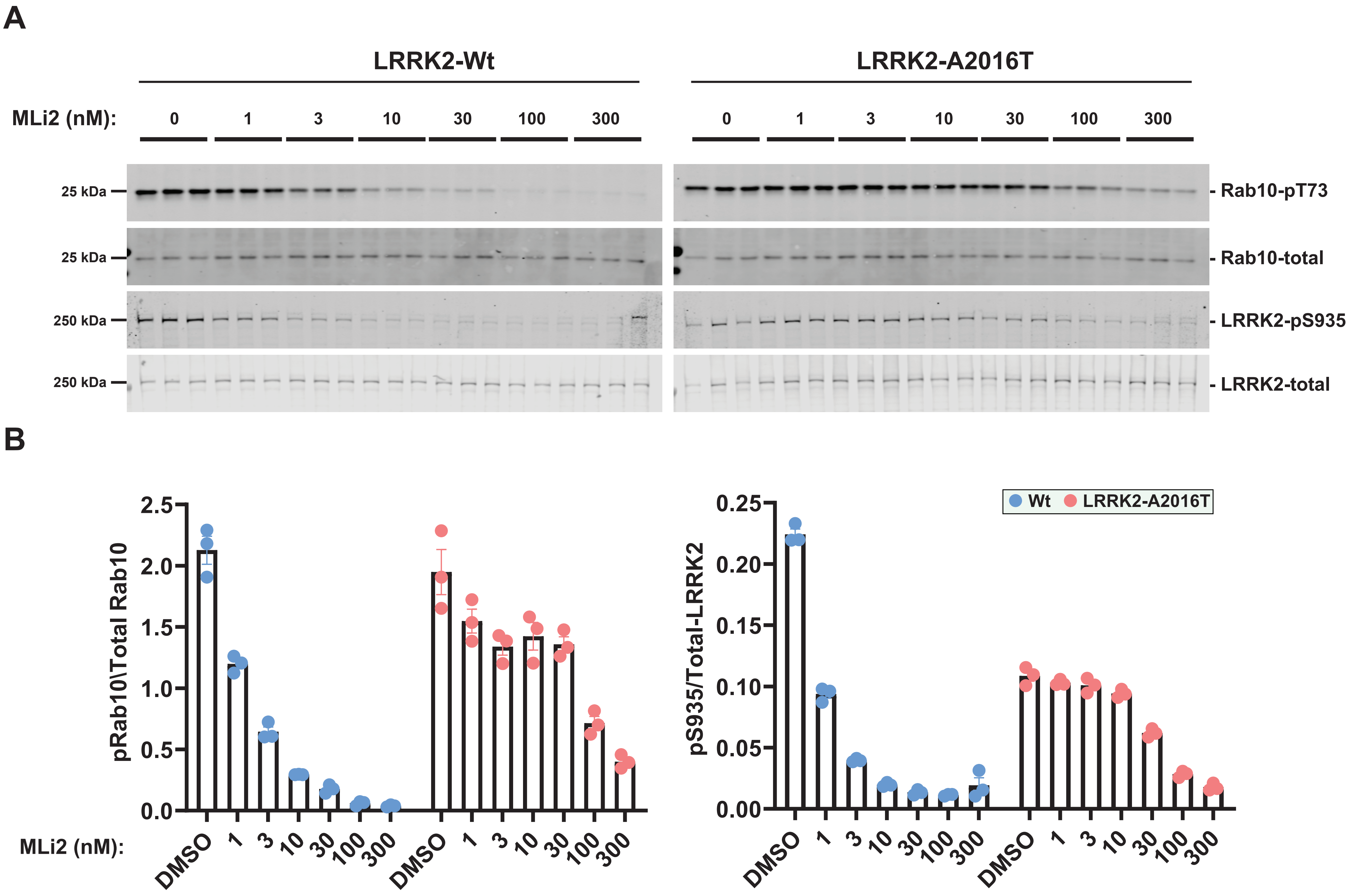

Supplementary Figure: 10

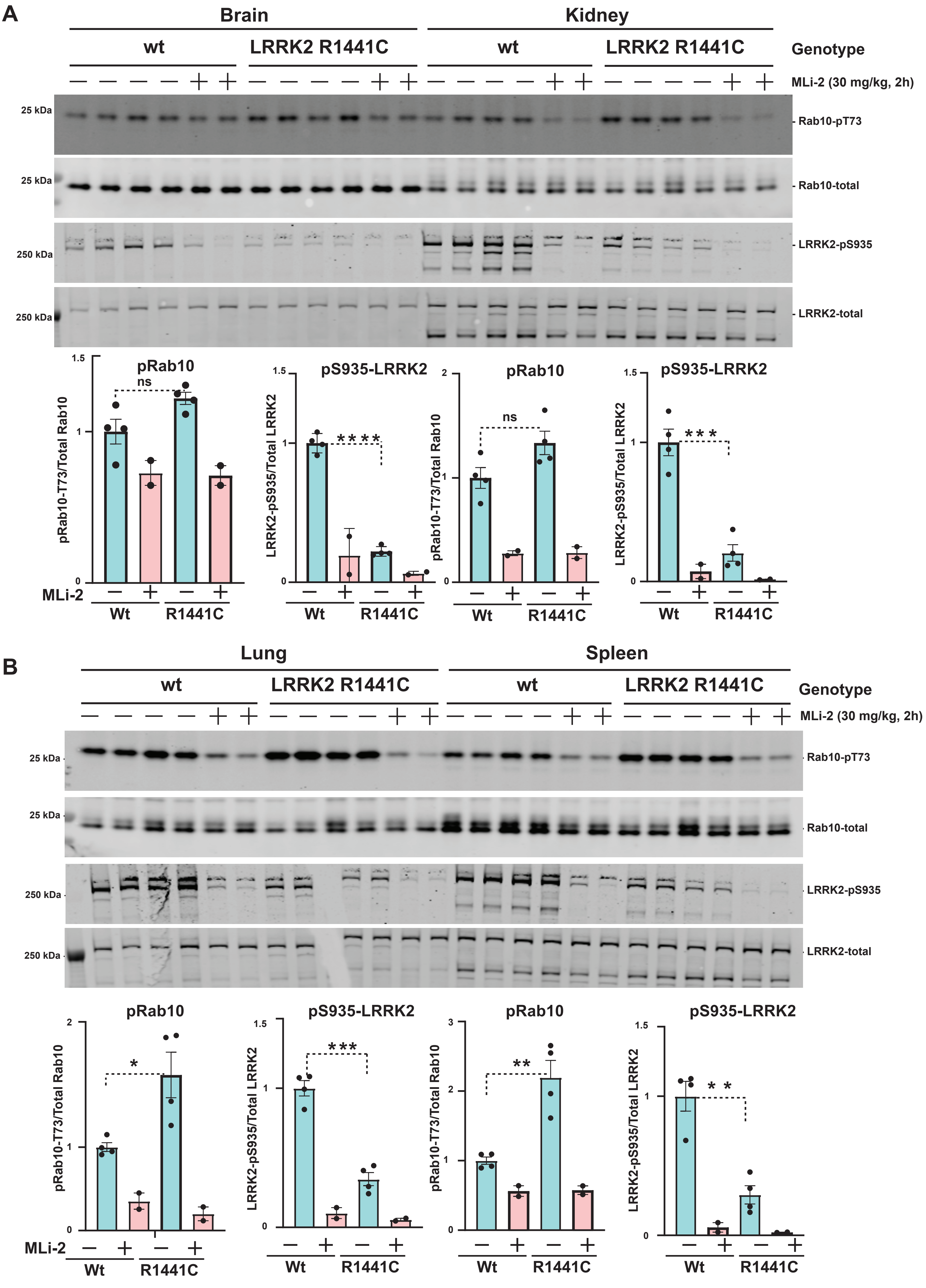

Supplementary Figure: 11

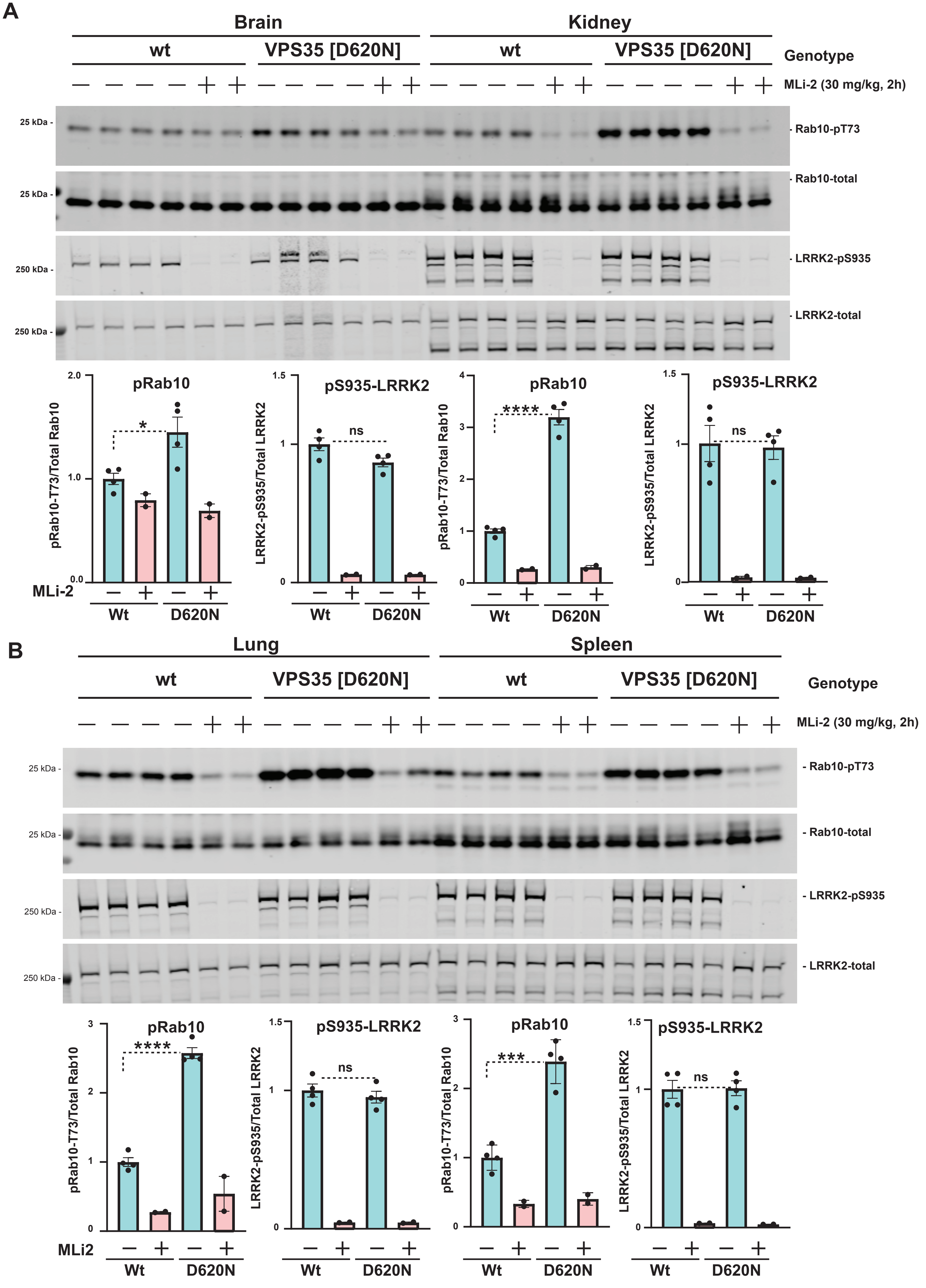

Supplementary Figure: 12

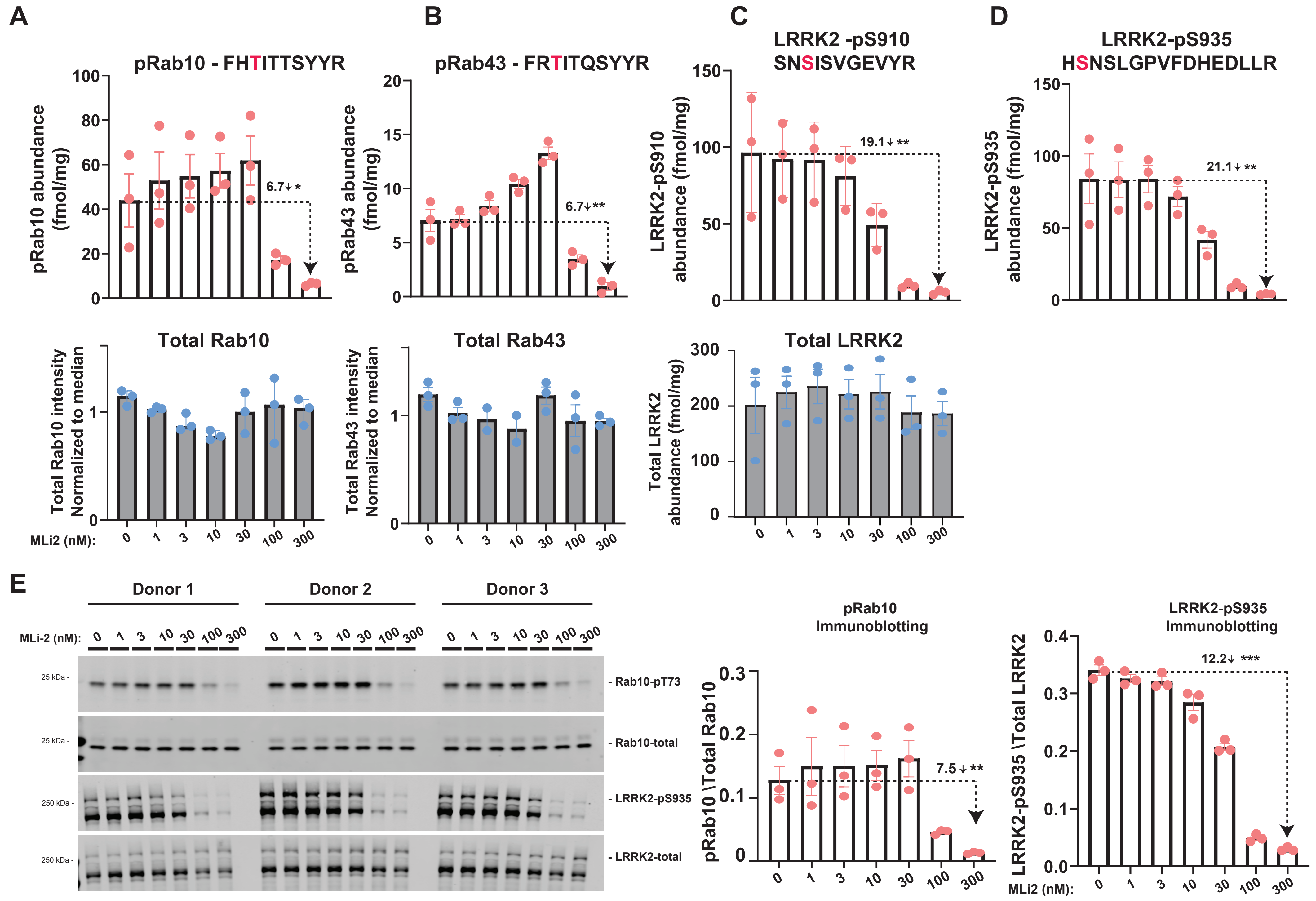

Supplement: Supplementary Figures S1-S12 [file BCJ-478-299-s1.pdf]
